# Fingerprint evidence for the division of labour and learning pottery-making at Early Bronze Age Tell eṣ-Ṣâfi/Gath, Israel

**DOI:** 10.1371/journal.pone.0231046

**Published:** 2020-04-17

**Authors:** Kent D. Fowler, Jon Ross, Elizabeth Walker, Christian Barritt-Cleary, Haskel J. Greenfield, Aren M. Maeir

**Affiliations:** 1 Department of Anthropology Ceramic Technology Laboratory, The University of Manitoba, Winnipeg, Canada; 2 St. John’s College, The University of Manitoba, Winnipeg, Canada; 3 Department of Anthropology, The University of Manitoba, Winnipeg, Canada; 4 St. Paul’s College, Near Eastern Biblical Archaeology Laboratory, The University of Manitoba, Winnipeg, Canada; 5 The Institute of Archaeology, The Martin (Szusz) Department of Land of Israel Studies and Archaeology, Bar-Ilan University, Ramat Gan, Israel; University at Buffalo - The State University of New York, UNITED STATES

## Abstract

The organization of craft production has long been a marker for broader social, economic and political changes that accompanied urbanism. The identity of producers who comprised production groups, communities, or workshops is out of reach using conventional archaeological data. There has been some success using epidermal prints on artefacts to identify the age and sex of producers. However, forensic research indicates that a combination of ridge breadth and ridge density would best identify the age and sex of individuals. To this end, we combine mean ridge breadth (MRB) and mean ridge density (MRD) to distinguish the age and sex of 112 fingerprints on Early Bronze Age (EB) III pottery from the early urban neighbourhood at Tell eṣ-Ṣâfi/Gath, Israel, dating to a 100 year time span. Our analysis accounts for the shrinkage of calcareous fabrics used to make six type of vessels, applies a modified version of the Kamp et al. regression equation to the MRB for each individual print, and infers sex by correlating MRD data to appropriate modern reference populations. When the results are combined, our analyses indicate that most fingerprints were made by adult and young males and the remainder by adult and young females. Children’s prints are in evidence but only occur on handles. Multiple prints of different age and sex on the same vessels suggest they were impressed during the training of young potters. Production appears dominated by adult and young males working alone, together, and in cooperation with adult and/or young females. Vessels with prints exclusively by females of any age are rare. This male dominant cooperative labour pattern contrasts recent studies showing that adult women primarily made Neolithic figurines in Anatolia, and more females than males were making pottery prior to the rise of city-states in northern Mesopotamia.

## Introduction

Forensic research has long established that epidermal prints are consistently different in ridge breadth and ridge density as a respective result of age and sexual dimorphism. Prints that survive on vessels, figurines and other ceramic objects hold great potential for providing insights into labour organization and how the craft was taught and learned, as they provide the only direct source of evidence for the demographics of ancient communities of potters [1].

It is not known if prints are actually rare on ceramics [2]. The preservation of prints likely results from a complex mix of factors: the use of surface finishing or decorative treatments, how objects were handled during manufacture, whether potters noticed or cared whether prints were on vessels, how objects were used, and a range of post-depositional conditions [3]. During manufacture, prints can be obliterated by wiping, smoothing, burnishing, polishing, painting, plastering or glazing wares, and are easily wiped off before an object has dried. Prints on commonly used objects, such as cooking, serving, and eating vessels, can be worn through wear, while those on rarely handled objects, such as storage vessels or sculpture, have a better chance of being preserved. The presence of prints on pottery is one of chance and the attention a potter cared to give them, but epidermal prints do occur on ancient ceramics. While they may not be rare, they are rarely studied despite consistent research by forensic scholars on developing and refining methods of identifying the age and sex of individuals from epidermal finger and palm prints [4–9].

Since Kamp et al.’s [10] seminal study introduced mean ridge breadth measurements to estimate the age of past printmakers, there has been sporadic attention to epidermal prints on archaeological ceramics. The efforts of Králík and colleagues marks much of the work during the 2000s [4, 11–13]. These studies provided more accurate estimations of age and an empirical basis to infer age and sex for single prints using the ridge breadth method. The initial work on ridge breadth led Králík, Novotný and Martin [12] to attempt aging the fingerprint on the Gravettian-age (c. 25,000 BCE) Dolní Věstonice figurine, resulting in what they considered an unlikely estimate of 7–15 yrs.

Subsequent work by Stinson [14] used a different method—ridge density—to examine the sex of printmakers on Hohokam figurines in the American southwest. Based on ridge counts in a one centimetre square area, she concluded that almost 80% of the figurines were made by women. By the mid- to late 2000s, Gungadin [15] was demonstrating a consistent relationship between ridge density and sexual dimorphism using a protocol that calculated ridge density in a 25 mm^2^ area. The benefit of this method is that was more easily applied to partial prints. It was only later that Sanders [16] again raised the profile of palaeodermatoglyphic research in his application of the ridge density method proposed by Gungadin to infer the sex of 106 prints on 101 vessels from Tell Leilan in northern Mesopotamia (Syria) spanning a period of some 2300 years (4100–1726 BCE). Sanders found that both male and female prints occur on vessels prior to the rise of the state mid-third millennium BCE, but only the prints of men occur on pottery in the post-state ceramics he examined; thus, he concluded that women ceased making pottery after the rise of city-states. The latter conclusion is problematic because the “post-state” sample involves 11 prints from sherds at five rural sites spanning over 870 years (2600–1726 BCE). This is neither representative of an early urban context nor is it an adequate sample for the time span involved. More recently, Bennison-Chapman and Hager [17] applied new imaging methods to palm and fingerprints on Neolithic clay “tokens” from Boncuklu Höyük in Turkey. Using ridge density, they found that adult women and men and children applied prints to the objects, but adult women left prints on tokens six times more often than men or children. A hanging concern with that analysis is that the authors did not consider how shrinkage may affect their results. Most recently, Kantner et al. [18] also utilized the ridge density method to sex prints found on an extremely large sample of corrugated vessels from six habitation clusters in the Blue J Ancestral Puebloan community in the American Southwest. They found that more women produced prints in the eastern clusters and more men produced prints in the western clusters. Corrugated pottery production was therefore not strictly gendered from the 10th to 11th centuries AD, but the results challenge the assumption that women were alone responsible for the production of domestic goods, such as cooking and serving vessels.

The state of current research on ancient fingerprints presents a welcome resurgence of interest in the value of prints for reconstructing the demographics of potting communities and labour organization. However, all of these studies examined age or sex, but not both, and none considered the potential of prints to inform about who could have been responsible for certain manufacturing operations.

Our recent study [3] reviewed methods of age determination and sex determination by forensic researchers and archaeologists to examine fingerprints on sherds from Early Bronze (EB) III (c. 2850–2500 BCE) levels in the urban neighbourhood at Tell eṣ-Ṣâfi/Gath in Israel. For age determination, we explored the further potential of Králík and Novotný’s data set [4]. We argued that Králík and Novotný’s robust sample makes it clear that ridge breadth <0.37 mm most clearly corresponds to prepubescent children while values >0.52 mm correlate with adult males. Values between these cannot confidently distinguish adults from adolescents. In part, this is complicated by the variable onset of puberty, which can range from 7–14 years [19] and because fingers stop growing in length around 15 years of age [20]. However, fingers can increase in width and thickness after maximum hand length has been achieved, as tendons will increase in density and size due to chronic resistance from intensive, repetitive activities [21], such as potting. Ridge breadth values in the adult range for early adolescents result from rapid growth spurts that can make adolescents indistinguishable from adults until the 0.52 mm threshold. Thus, we proposed that only three definitive age/sex categories that can be inferred from ridge breadth measures: adult males, adult/adolescents, and prepubescent children under the age of 10.

In regard to sex determination, we considered biological affinity and studies that measured radial ridge density to propose a population reference sample that included data from only Turkish and Indian populations. The inclusion of datasets from Thai, Chinese, Filipino, African, or African American populations could not be justified because Neolithic and EB populations share greater genetic relatedness to central Asian, north-east African, and European populations [22]. The resulting model suggested >95% probability thresholds of ridge density data values <12.99/25mm^2^ for men and >15.60/25mm^2^ for women.

Next, following the suggestions of forensic researchers, such as Soanboon et al. [23], we introduced an *identification matrix* as an interpretive framework that incorporates the generalized >95% probability thresholds for age based upon ridge breadth data and the >95% probability thresholds for sex based upon ridge density data. The matrix improves the estimate of age and sex determination for prints of unknown origin and more clearly distinguishes adolescent and adult male and female prints, allowing prints to be classified into one of six age/sex categories and not the three categories that can be inferred from ridge breadth data alone.

Applying these methods to a small sample of high-quality prints on EB III pottery at Tell eṣ-Ṣâfi/Gath, we concluded that “pottery production was not restricted to one gender during the EB III and production was organized with adolescent cooperative labour” [3]. Evidence for the inclusion of children in the manufacturing process was inconclusive. Our observation of print locations, manufacturing traces on sherds, and our age/sex estimates of prints led us to infer when the prints were impressed on the surface of vessels and who handled vessels during manufacture. In sum, each class of cooking and storing vessels had different hands on them during and after shaping: only men made bowls, only men and adolescent males made cooking vessels, women and adolescent girls more often made large storage jars, and both males and females made smaller, wavy-handled storage jars.

### Hypotheses

Although our previous sample was smaller than most of the studies mentioned above, it had the advantages of using only extremely high-quality prints restricted to a short time frame (100 years). Here, we formulate the trends observed in our methodological study as hypotheses to test against a larger sample dating from EB III levels at Tell eṣ-Ṣâfi/Gath to better understand the social context of ceramic production during this period of profound social change in the Levant. The hypotheses we propose include:

1. Males and females made different kinds of pottery in the EB III repertoire.
2. The labour force used to make EB III pottery involved men, women, and teenagers of either sex.
3. Multiple hands were normally involved in vessel shaping and adults and adolescents had different roles in manufacture.
4. A greater proportion of adolescent boys learned to be potters and practiced the craft while adults, but fewer adolescent girls continued to make pottery into adulthood.
5. A larger sample size would confirm more males than females were involved in potting, but a larger proportion of prints would be identified to adult females and individuals of adolescent age.

In the following, we outline the sample and our analytical methods, report the results of our age and sex analyses, and conclude with a discussion of the hypotheses as a stimulus for using epidermal prints in efforts to compare and explain the relationship between the organization of crafts production and forms of socio-political organization within and beyond the Near East.

## Materials

The EB II–III (c. 3100–2500 BCE) is the beginning of urbanism and burgeoning (likely secondary) state societies in the Levant. Settlement size and density increases across the Levant resulting a regional hierarchy with several levels, including fortified cities and towns, large and small villages dispersed between them, and transitory settlements (camps and cave-dwellings) [24–28]. The appearance of urban centers, palaces (such as at Yarmuth) [29], and various administrative activities indicated by the use of glyptic devices [27, 30, 31] has been used to propose that city-states begin to emerge during the period. There are clear indications of predetermined planning attested by well-defined housing blocks, street networks, industrial spaces, storage facilities, civic buildings, and organised public spaces at a variety of usually fortified towns and cities (e.g., Eshtaol, Qyriat Ata, Erani, Assawir, Beth Yerah, Ashkelon, and Tel Megiddo East).

**Fig 1:**
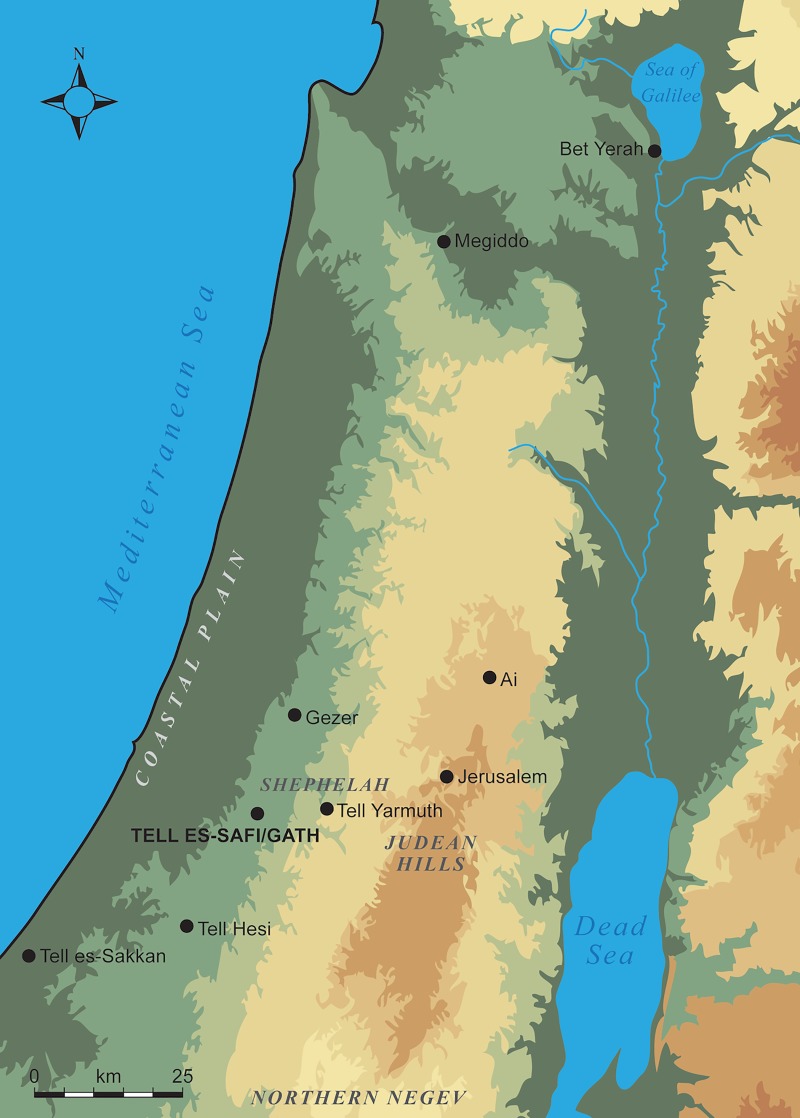
Map of the region showing the location of Tell eṣ-Ṣâfi/Gath and other relevant EB sites, regions and features in the southern levant.

Tell eṣ-Ṣâfi/Gath (Fig 1) is one such settlement. It is located at the western edge of the *shephelah* (Judean foothills) and overlooks the southern coastal plain of Israel [32–37]. It was occupied intermittently from the Chalcolithic (5^th^-4^th^ mill. BCE) through to the 20^th^ century CE. During the EB III, it was one of the paramount (fortified urban centres) sites in the regional settlement system across the southern Levantine landscape. Excavations have demonstrated that the EB settlement reached its maximum extent of 24 ha across the entire “upper mound” [38, 39] and was surrounded by a massive fortification system [40]. At the eastern end of the site, excavations have uncovered the remains of a substantive EB III domestic neighbourhood. All the material remains within this stratum appear to be related to domestic household consumption of food and other goods. The pottery remains used in this analysis derive from the final EB III occupation in this neighbourhood (i.e., Stratum E5) that was continuously occupied over a 100-year period dating from c. 2700–2600 BCE, based on stratigraphic analysis and the pottery assemblage [35, 41, 42]. The regional positioning, size, layout, fortifications, and evidence for administrative activities [41, 43], suggest that Tell eṣ-Ṣâfi/Gath is an urban centre during this period and arguably home to a local city-state.

Our sample consists of over 400 pottery sherds representing as many vessels from EB III levels. At this point, we have identified 57 sherds from different vessels that have 150 complete or partial fingerprint ridges. Of this set, we originally selected 18 sherds from as many vessels and examined 38 of the clearest partial or whole prints resulting from the impression of distal phalanxes on the interior and exterior of vessel surfaces at the plastic or leather-hard stages of drying, or on plaster during the post-firing treatment of vessels [3]. In the present analysis, we examine 115 partial fingerprints from 47 distinct vessels (Table 1). This sample more than doubles our original print and vessel sample sizes. We could not include the 35 other prints in this study because they had too few ridges, were obscured by later, overlapping prints, were damaged and not clear enough to be measured, or were placed on severely convex or concave surfaces (e.g. handles). Within the expanded subsample, prints occur primarily on body sherds from six classes of vessels: two kinds of storage jars, holemouth cooking vessels, bowls, jugs and juglets [39]. All of these vessel types were made by coiling [44].

**Table 1:** Contextual and excavation data for the fingerprint sample.

Analysis No.	Square/Unit	Locus	Basket	Stratum	Building No.	Definition	Vessel class	Fabric type	Location of print	Print type
17–100	83A	19E83A05	19E83A 017	E5c	16E83A10	Floor accumulation	Jar	Group 3	Exterior, base	Relief
17–101	73D	16E73D01	16E73D001	n/a	n/a	Winter wash	Jar	Group 3	Interior, body	Surface
17–116	83C	P	19E83C007	n/a	n/a	Winter wash	Holemouth	Group 2	Exterior, upper body	Surface
17–119	83A	E15AL03	E15AL009	E5a	94413	Floor accumulation	Holemouth	Group 2	Exterior, base	Surface
17–124	73D-83B	74808	748129	E5a	74808	Occupational debris	Jar	Group 3	Exterior, body	Surface
17–126	93A	19E93A07	19E93A 063	E7	n/a	Probe	Holemouth	Group 2	Exterior, body	Relief
17–133	83C	17E83C02	17E83C022	E5B	74512	Occupational debris	Jar	Group 3	Exterior, body	Surface
17–137	83A	19E83A08	19E83A100	E5C-E6	n/a	Probe	Jar	Group 3	Interior, body	Surface
17–142	93A	18E93A01	18E93A008	n/a	n/a	Winter wash	Holemouth	Group 2	Interior, upper body	Surface
17–143	83B	19E83B03	19E83B016	E5c	n/a	Baulk dismantle	Holemouth	Group 2	Interior, body	Surface
17–145	83B	19E83B03	19E83B052	E6	n/a	Probe	Holemouth	Group 2	Interior, upper body	Surface
17–146	83D	19E83D01	19E83D001	n/a	n/a	Winter wash	Holemouth	Group 2	Exterior, body	Surface
17–148		94105	941020	E4a	n/a	LB Wall	Wavy handled jar	Group 3	Interior, body	Surface
17–149	93B	114406	1144028	E5b	84309	Floor accumulation	Wavy handled jar	Group 3	Interior, body at handle	Surface
17–150		114808	1148024–1	Eb5	n/a	Fill?	Holemouth	Group 2	Interior, body	Relief
17–151	82D	144906	1449046	E5a	104311	Floor accumulation	Wavy handled jar	Group 3	Exterior, lower body	Relief
17–152	83D	E15AR10	E15AR096	E4-5	n/a	Fill?	Bowl	Group 3	Interior, rim	Surface
17–153	84C	E15AS02	E15AS044	E4-5	n/a	Fill	Jar	Group 3	Exterior, body	Surface
18–101		20E93A06	149	E7	n/a	E7 bulding collapse	Holemouth	Group 2	Interior, body	Surface
18–102	83D	20E83D04	81	E6	n/a	Floor make-up	Holemouth	Group 2	Interior, body	Surface
18–103	84C	20E84C03	C065	E6	n/a	Decayed brick debris east of WE15AS12	Holemouth	Group 2	Indeterminate, body	Surface
18–104	93A	18E93A01	18E93A0?	E5	n/a	Winter wash	Holemouth	Group 2	Interior, rim	Surface
18–105	93B	20E93B03	34	E6	n/a	Floor make-up	Holemouth	Group 2	Indeterminate, body	Surface
18–106	93A	20E93A03	93A148	E6(?)	n/a	Layer containing the donkeys sealed by E5c floor	Closed vessel	Group 3	Indeterminate, body	Surface
18–107	83D	20E83D02	D023	E6	n/a	Fill layer abuts the N face of WE15AS11	Jar	Group 3	Exterior, body	Surface
18–108	83C	20E83C01	1		n/a	Winter wash	Closed vessel	Group 3	Indeterminate, body	Surface
18–109	83A	19E83A07	A021	E5c	16E83A10	Brown soil abuts wall 17E83A11	Jar	Group 3	Interior, body	Surface
18–110		19E83A07	62		16E83A10	Brown soil abuts wall 17E83A11	Holemouth	Group 2	Exterior, body	Surface
18–111		144800	1448002		n/a	Winter wash	Closed vessel	Group 3	Indeterminate, body	Surface
18–112	83c	20E83C07	63	E5c	134307	Floor 18E83C09, ashy layer, cobbles	Jar	Group 3	Interior, body	Surface
18–113	93A	19E93A04	2	E5c	17E82D08	E5c floor, construction fill for E5 building	Holemouth	Group 2	Interior, base	Surface
18–114	83D	20E83D06	78	E7	Not assigned	Floor of stone platform 20E83D09	Holemouth	Group 2	Indeterminate, body	Surface
18–115	83D	20E83D07	176	E7	Not assigned	Ash layer, floor of stone platform 20E83D09	Closed vessel	Group 3	Indeterminate, body	Surface
18–116	31A	P15AG03	14		n/a	EB III sherd, east fortification wall	Juglet	Group 3	Interior, body	Surface
18–117	52A	P15AJ03	10		n/a	EB III sherd, east fortification wall	Wavy handled jar	Group 3	Exterior, handle	Linear
18–118	83D	20E83D05	92	E7	Not assigned	Ash layer, floor of stone platform 20E83D09	Holemouth	Group 2	Exterior, body	Surface
18–119	93A	104103	1041006	E5	n/a	Leveling square	Holemouth	Group 2	Indeterminate, body	Surface
18–120	73D	104803	1048033	E5b	114805	Winter wash	Jar	Group 3	Interior, body	Surface
18–121	83B	134307	1343131	E5c	134307	Floor	Jug	Group 3	Interior, body	Surface
18–122	84C	E15AS04	S057	E4		Intrusive EB III sherd on LB wall	Jar	Group 3	Handle	Surface
18–123	93B	114406	1144028	E5b	84309	Floor accumulation	Wavy handle jar	Group 3	Exterior, handle	Surface
18–124		94105	941020	E4a		Intrusive EB III sherd in fill layer with bricks and mixed EB III and LB material	Jar	Group 3	Interior, handle and body	Surface
18–125	93A	19E93A04	5	E5c	17E82D08	E5c floor make-up and construction fill for the E5 building	Jug	Group 3	Interior, body	Surface
18–126		E15AS02	AS044	E4-E5	n/a	EB III sherd in fill layer with bricks and mixed LB and EB remains.	Jar	Group 3	Exterior, body	Surface
18–127	93A	19E93A04	A006	E5c	17E82D08	E5c floor make-up and construction fill for the E5 building	Jug	Group 3	Interior, body	Surface
18–128	93A	104105	1041011	E4-E5	n/a	Stone wall foundations for the SE corner of building 104311	Holemouth	Group 2	Interior, rim	Surface
18–129	83B	19E83B03	19E83B016	E5c	134307	Floor	Holemouth	Group 2	Indeterminate, body	Surface

## Methods

Once prints were identified, their relative location on the vessel form was recorded and they were classified into one of three categories. Fingerprints occur on both the exterior and interior surfaces of all closed vessel forms (holemouth, jar, wavy handled jar, jug, and juglet), and only on the interior of the open bowl (Table 1). Each print was classified by one of three ways that fingers were applied on vessels [3]: (1) *surface prints* result from fingers being applied directly to the surface of the vessel; (2) *relief prints* result from a dab of clay or plaster impressed on the surface with a clear impression of the print; and (3) *linear prints* are when print extends across the vessel surface, implying a wiping gesture involving a finger or thumb edge along the surface resulting in a partial “extended” print that has the same ridge and valley breadth and ridge density patterns as surface or relief prints. All three categories of prints are distinctively different from patterns left by implements, such as cloth, reeds or hard tools, used to finish vessel surfaces. Prints on both the interior and exterior of vessels were placed after the surface had been scraped, wiped or smoothed.

Our methods of image acquisition and print analysis follow those described in Fowler et al. [3]. In brief, prints on the sherds and dental moulds of the prints were scanned at 600 dpi on a flatbed scanner and photographed on a flat table top using a digital SLR camera set perpendicular to the print and centred in the frame next to a metric scale in millimeters [cf. 4]. Dental moulds invert ridges and furrows. They often allow clearer observations of prints because the moulds remove sherd colour and/or grit inclusions from a visual assessment, as these can obscure boundaries of multiple or overlapping prints, and interrupt ridge-furrow pairs and groups, making measurements more difficult. All images were imported into Photoshop® and enhanced by adjusting the image contrast and exposure. The adjusted images were then uploaded into the program Macnification® and calibrated for measurement.

Our calculation of age and sex estimates considers the shrinkage of clay during drying and firing [3]. A failure to account for shrinkage will skew age and sex estimates. Ridge breadth *decreases* with shrinkage so age will be underestimated. Ridge density *increases* with shrinkage so sex determination will be skewed towards females if shrinkage rates are not considered. Local marly or calcareous tempered clays were used to make the EB pottery represented in our sample. These fall into two major fabric groups (Table 1). Group 2 are clays derived from dark brown loess soils with abundant, poorly sorted calcareous inclusions up to 2 mm in length, and minor amounts of limestone, calcite, calcrete (*nari*), and sandstone (eolianite or *kurkar*). Group 3 clays derived from rendzina soils and are characterized by naturally occurring rounded silt to medium sand-sized chalk and limestone inclusions, angular grog temper, burned out organics, and microfossils [45, 46]. The fabric types vary by vessel function: Group 2 is for cooking wares, and Group 3, the most common fabric type, is used for coarse ware jars, jugs, juglets, and bowls [3]. Raw marly clays will undergo 2–6% linear shrinkage when fired [47–50]. The inclusion of calcareous and other gritty materials would only act to decrease the maximum natural shrinkage of the raw clay.

The Mean of Ridge-Furrow Pairs Ridge Breadth (MPRB) method was used to estimate age and we modified Kamp et al.’s [10] original regression equation to account for the particular linear shrinkage range (2–6%) experienced by marly clays when fired. We refer to this as the KAmod2 equation so as not to confuse it with Králík and Novotný’s [4] KAmod formula. Thus, the formula used to arrive at the age estimates in Table 2 is KAmod2: Age (mo) = 614 · MRB (mm)– 112 · (shrinkage: 0.02 or 0.06). Ridge breadth values *decrease* due to linear shrinkage (the ridge-furrow breadth narrows), so our calculation *increased* the values by 2% and 6%. The Kamp et al. error range of ±2.25 years was then applied to each midpoint to arrive at a total error range for each age estimate of a print that was adjusted for clay shrinkage. The two prints in our sample were impressed on plaster and did not experience linear shrinkage (17–100:A, B).

**Table 2:** Application of KAmod2 regression equation and ridge density threshold (see text) to the sample of prints.

Cat. No.	Print No.	Mean Ridge Breadth, Age/Sex Category, Age Range^1^								Mean Ridge Density and Sex Classification				Matrix Classification^2^	
		Avg. MRB 2%	Avg. Age (yrs) 2%	Age/Sex Cat.	Avg. MRB 6%	Avg. Age (yrs) 6%	Age/Sex Cat.	Min. Age (yrs)	Max. Age (yrs)	MRD 2%	MRD 2% Sex	MRD 6%	MRD 6% Sex	2%	6%
**17–100**	17–100:A	0.50	16.15	AA	0.52	17.15	AM	13.40	18.40	11.00	M	11.00	M	AAM	AM
	17–100:B	0.47	14.66	AA	0.49	15.60	AA	11.94	16.91	12.00	M	12.00	M	AAM	AAM
**17–101**	17–101:A	0.44	13.34	AA	0.46	14.23	AA	10.65	15.59	13.72	F	13.16	F	AAF	AAF
	17–101:C	0.46	14.30	AA	0.48	15.22	AA	11.58	16.55	15.68	F	15.04	F	AAF	AAF
	17–101:D	0.40	11.28	AA	0.42	12.09	AA	8.63	13.53	15.68	F	15.04	F	AAF	AAF
	17–101:E	0.50	16.24	AA	0.52	17.24	AM	13.49	18.49	13.72	F	13.16	F	AAF	AF
**17–116**	17–116:A	0.52	17.37	AM	0.54	18.42	AM	14.60	19.62	11.76	M	11.28	M	AM	AM
**17–119**	17–119:A	0.52	17.48	AM	0.54	18.53	AM	14.70	19.73	9.80	M	9.40	M	AM	AM
	17–119:B	0.46	14.33	AA	0.48	15.25	AA	11.61	16.58	7.84	M	7.52	M	AAM	AAM
**17–124**	17–124:A	0.54	18.43	AM	0.56	19.52	AM	15.64	20.68	13.72	F	13.16	F	AF	AF
	17–124:B	0.52	17.46	AM	0.54	18.51	AM	14.68	19.71	11.76	M	11.28	M	AM	AM
	17–124:C	0.51	16.57	AA	0.53	17.58	AM	13.81	18.82	15.68	F	15.04	F	AAF	AF
**17–126**	17–126:A									11.76	M	11.28	M	M	M
	17–126:C	0.63	22.70	AM	0.65	23.95	AM	19.82	24.95	9.80	M	9.40	M	AM	AM
	17–126:D	0.41	11.83	AA	0.43	12.66	AA	9.17	14.08	10.78	M	10.34	M	AAM	AAM
	17–126:E	0.66	24.66	AM	0.69	25.99	AM	21.74	26.91	11.76	M	11.28	M	AM	AM
	17–126:F	0.37	9.72	AA	0.39	10.46	AA	7.09	11.97	8.82	M	8.46	M	AAM	AAM
**17–133**	17–133:A	0.60	21.20	AM	0.62	22.40	AM	18.35	23.45	11.76	M	11.28	M	AM	AM
	17–133:B	0.62	22.18	AM	0.64	23.41	AM	19.31	24.43	11.76	M	11.28	M	AM	AM
	17–133:C	0.56	19.47	AM	0.59	20.60	AM	16.66	21.72	11.76	M	11.28	M	AM	AM
	17–133:D	0.71	26.85	AM	0.73	28.27	AM	23.89	29.10	9.80	M	9.40	M	AM	AM
	17–133:F									13.72	F	13.16	F	F	F
**17–137**	17–137:A	0.58	20.24	AM	0.60	21.40	AM	17.41	22.49	13.72	F	13.16	F	AF	AF
	17–137:B	0.58	20.15	AM	0.60	21.31	AM	17.33	22.40	11.76	M	11.28	M	AM	AM
**17–142**	17–142:A	0.36	9.30	CH	0.38	10.03	AA	6.68	11.55	11.76	M	11.28	M	CHM	AAM
**17–143**	17–143:A	0.51	16.52	AA	0.53	17.53	AM	13.76	18.77	10.78	M	10.34	M	AAM	AM
	17–143:B	0.52	17.28	AM	0.54	18.33	AM	14.51	19.53	10.78	M	10.34	M	AM	AM
	17–143:C									6.86	M	6.58	M	M	M
	17–143:D									8.82	M	8.46	M	M	M
**17–145**	17–145:D	0.50	16.45	AA	0.52	17.46	AM	13.69	18.70					AA	AM
**17–146**	17–146:D	0.64	23.35	AM	0.66	24.63	AM	20.46	25.60					AM	AM
**17–148**	17–148:A	0.40	11.00	AA	0.41	11.79	AA	8.35	13.25	15.68	F	15.04	F	AAF	AAF
	17–148:B	0.50	16.12	AA	0.52	17.12	AM	13.37	18.37	10.78	M	10.34	M	AAM	AM
**17–149**	17–149:A	0.55	18.85	AM	0.57	19.95	AM	16.04	21.10	11.76	M	11.28	M	AM	AM
**17–150**	17–150:A	0.53	18.01	AM	0.56	19.08	AM	15.23	20.26	11.76	M	11.28	M	AM	AM
**17–151**	17–151:A	0.54	18.40	AM	0.56	19.49	AM	15.61	20.65	13.72	F	13.16	F	AF	AF
**17–152**	17–152:A	0.56	19.18	AM	0.58	20.30	AM	16.38	21.43	11.76	M	11.28	M	AM	AM
**17–153**	17–153:A	0.40	11.24	AA	0.42	12.04	AA	8.58	13.49	12.74	M	12.22	M	AAM	AAM
	17–153:B	0.40	11.23	AA	0.42	12.04	AA	8.58	13.48	15.68	F	15.04	F	AAF	AAF
	17–153:C	0.50	16.50	AA	0.52	17.51	AM	13.74	18.75	15.68	F	15.04	F	AAF	AF
**18–100**	18–100:A	0.60	21.22	AM	0.62	22.42	AM	18.37	23.47	11.76	M	11.28	M	AM	AM
	18–100:B	0.62	22.58	AM	0.65	23.83	AM	19.70	24.83	14.21	F	13.63	F	AF	AF
	18–100:C	0.37	9.60	AA	0.38	10.34	AA	6.98	11.85	12.74	M	12.22	M	AAM	AAM
**18–102**	18–102:A	0.61	22.06	AM	0.64	23.29	AM	19.19	24.31	11.76	M	11.28	M	AM	AM
	18–102:B	0.62	22.49	AM	0.65	23.73	AM	19.61	24.74	13.07	F	12.53	M	AF	AM
**18–103**	18–103:A	0.60	21.16	AM	0.62	22.35	AM	18.31	23.41	15.03	F	14.41	F	AF	AF
	18–103:B	0.37	9.82	AA	0.39	10.57	AA	7.19	12.07	17.64	F	16.92	F	AAF	AAF
**18–104**	18–104:A	0.42	12.04	AA	0.43	12.88	AA	9.37	14.29	14.70	F	14.10	F	AAF	AAF
	18–104:B	0.41	11.78	AA	0.43	12.60	AA	9.11	14.03	14.95	F	14.34	F	AAF	AAF
**18–105**	18–105:A	0.56	19.28	AM	0.58	20.40	AM	16.47	21.53	13.02	F	12.49	M	AF	AM
**18–106**	18–106:A	0.65	24.09	AM	0.68	25.40	AM	21.18	26.34	7.84	M	7.52	M	AM	AM
**18–107**	18–107:A	0.48	15.18	AA	0.50	16.14	AA	12.45	17.43	9.80	M	9.40	M	AAM	AAM
	18–107:B	0.56	19.49	AM	0.59	20.62	AM	16.67	21.74	12.54	M	12.03	M	AM	AM
**18–108**	18–108:A	0.66	24.57	AM	0.69	25.90	AM	21.66	26.82	7.84	M	7.52	M	AM	AM
	18–108:B	0.50	16.24	AA	0.52	17.24	AM	13.49	18.49	9.80	M	9.40	M	AAM	AM
	18–108:C	0.53	17.72	AM	0.55	18.79	AM	14.94	19.97	12.74	M	12.22	M	AM	AM
	18–108:D	0.45	13.64	AA	0.47	14.54	AA	10.94	15.89	14.70	F	14.10	F	AAF	AAF
	18–108:E	0.61	21.88	AM	0.63	23.10	AM	19.01	24.13	10.78	M	10.34	M	AM	AM
	18–108:F	0.42	12.20	AA	0.44	13.04	AA	9.52	14.45	13.72	F	13.16	F	AAF	AAF
	18–108:G	0.55	18.85	AM	0.57	19.95	AM	16.05	21.10	9.80	M	9.40	M	AM	AM
**18–109**	18–109:A	0.53	17.96	AM	0.55	19.03	AM	15.17	20.21	12.41	M	11.91	M	AM	AM
	18–109:B	0.76	29.34	AM	0.79	30.85	AM	26.33	31.59	7.84	M	7.52	M	AM	AM
	18–109:C	0.64	23.48	AM	0.67	24.76	AM	20.58	25.73	10.78	M	10.34	M	AM	AM
	18–109:D	0.57	20.08	AM	0.60	21.24	AM	17.26	22.33	10.54	M	10.11	M	AM	AM
**18–110**	18–110:A	0.52	17.44	AM	0.54	18.49	AM	14.67	19.69	12.41	M	11.91	M	AM	AM
**18–111**	18–111:A	0.60	21.27	AM	0.62	22.47	AM	18.42	23.52	11.11	M	10.65	M	AM	AM
	18–111:B	0.73	28.25	AM	0.76	29.73	AM	25.27	30.50	10.78	M	10.34	M	AM	AM
**18–112**	18–112:A	0.61	21.70	AM	0.63	22.92	AM	18.84	23.95	11.43	M	10.97	M	AM	AM
	18–112:B	0.60	21.40	AM	0.62	22.61	AM	18.55	23.65	9.15	M	8.77	M	AM	AM
	18–112:C	0.63	23.15	AM	0.66	24.43	AM	20.27	25.40	9.80	M	9.40	M	AM	AM
**18–113**	18–113:A	0.39	10.87	AA	0.41	11.66	AA	8.22	13.12	13.72	F	13.16	F	AAF	AAF
	18–113:B	0.46	14.33	AA	0.48	15.26	AA	11.61	16.58	11.76	M	11.28	M	AAM	AAM
**18–114**	18–114:A	0.46	13.95	AA	0.47	14.86	AA	11.25	16.20	15.48	F	14.85	F	AAF	AAF
**18–115**	18–115:A	0.47	14.70	AA	0.49	15.64	AA	11.98	16.95	13.72	F	13.16	F	AAF	AAF
	18–115:B	0.51	17.01	AA	0.53	18.04	AM	14.24	19.26	11.76	M	11.28	M	AAM	AM
	18–115:C	0.50	16.36	AA	0.52	17.37	AM	13.61	18.61	12.74	M	12.22	M	AAM	AM
**18–116**	18–116:A	0.60	21.24	AM	0.62	22.44	AM	18.39	23.49	11.11	M	10.65	M	AM	AM
	18–116:B	0.52	17.03	AM	0.54	18.06	AM	14.26	19.28	10.78	M	10.34	M	AM	AM
	18–116:C	0.58	20.52	AM	0.61	21.69	AM	17.69	22.77	11.76	M	11.28	M	AM	AM
	18–116:D	0.45	13.60	AA	0.47	14.50	AA	10.90	15.85	11.76	M	11.28	M	AAM	AAM
	18–116:E	0.49	15.82	AA	0.51	16.81	AA	13.08	18.07	12.74	M	12.22	M	AAM	AAM
**18–117**	18–117:A	0.42	12.01	AA	0.43	12.85	AA	9.34	14.26	11.76	M	11.28	M	AAM	AAM
	18–117:B	0.73	28.16	AM	0.76	29.63	AM	25.17	30.41	5.39	M	5.17	M	AM	AM
	18–117:C	0.69	26.00	AM	0.72	27.39	AM	23.06	28.25	9.80	M	9.40	M	AM	AM
	18–117:D	0.43	12.85	AA	0.45	13.72	AA	10.16	15.10	14.70	F	14.10	F	AAF	AAF
	18–117:E	0.35	8.54	CH	0.36	9.24	CH	5.94	10.79					CH*	CH*
**18–118**	18–118:A	0.50	16.30	AA	0.52	17.31	AM	13.55	18.55	12.74	M	12.22	M	AAM	AM
**18–119**	18–119:A	0.50	16.02	AA	0.51	17.01	AA	13.27	18.27	11.76	M	11.28	M	AAM	AAM
**18–120**	18–120:A	0.42	11.91	AA	0.43	12.75	AA	9.25	14.16	16.33	F	15.67	F	AAF	AAF
**18–121**	18–121:A	0.52	17.30	AM	0.54	18.34	AM	14.52	19.55	15.68	F	15.04	F	AF	AF
	18–121:B	0.67	24.76	AM	0.69	26.10	AM	21.85	27.01	11.76	M	11.28	M	AM	AM
**18–122**	18–122:A	0.48	15.09	AA	0.50	16.05	AA	12.36	17.34	13.07	F	12.53	M	AAF	AAM
**18–123**	18–123:A	0.58	20.54	AM	0.61	21.71	AM	17.71	22.79	10.45	M	10.03	M	AM	AM
	18–123:B	0.56	19.37	AM	0.58	20.50	AM	16.56	21.62	11.76	M	11.28	M	AM	AM
	18–123:C	1.39	61.65	AM	1.44	64.43	AM	58.00	63.90	5.88	M	5.64	M	AM	AM
	18–123:D	0.85	34.18	AM	0.88	35.89	AM	31.08	36.43	7.84	M	7.52	M	AM	AM
**18–124**	18–124:A	0.36	9.19	CH	0.38	9.92	AA	6.58	11.44	9.80	M	9.40	M	CHM	AAM
	18–124:B	0.43	12.72	AA	0.45	13.58	AA	10.03	14.97	9.80	M	9.40	M	AAM	AAM
**18–125**	18–125:A	0.60	21.60	AM	0.63	22.82	AM	18.75	23.85	9.80	M	9.40	M	AM	AM
	18–125:B	0.61	21.63	AM	0.63	22.85	AM	18.78	23.88	7.84	M	7.52	M	AM	AM
	18–125:C	0.66	24.35	AM	0.68	25.67	AM	21.44	26.60	8.33	M	7.99	M	AM	AM
**18–126**	18–126:A	0.43	12.48	AA	0.44	13.33	AA	9.80	14.73	15.44	F	14.81	F	AAF	AAF
	18–126:B	0.43	12.46	AA	0.44	13.31	AA	9.78	14.71	15.35	F	14.73	F	AAF	AAF
	18–126:C	0.49	15.99	AA	0.51	16.99	AA	13.25	18.24	15.29	F	14.66	F	AAF	AAF
**18–127**	18–127:A	0.43	12.61	AA	0.45	13.47	AA	9.93	14.86	16.66	F	15.98	F	AAF	AAF
	18–127:B	0.40	11.31	AA	0.42	12.12	AA	8.66	13.56	12.74	M	12.22	M	AAM	AAM
**18–128**	18–128:A	0.51	16.91	AA	0.53	17.94	AM	14.15	19.16	13.72	F	13.16	F	AAF	AF
	18–128:B	0.49	15.76	AA	0.51	16.75	AA	13.02	18.01	10.78	M	10.34	M	AAM	AAM
**18–129**	18–129:A	0.62	22.24	AM	0.64	23.47	AM	19.37	24.49	9.15	M	8.77	M	AM	AM
	18–129:B	0.52	17.23	AM	0.54	18.27	AM	14.46	19.48	12.74	M	12.22	M	AM	AM
	18–129:C	0.59	20.84	AM	0.61	22.02	AM	18.00	23.09	13.07	F	12.53	M	AF	AM
	18–129:D	0.60	21.50	AM	0.63	22.71	AM	18.65	23.75	10.78	M	10.34	M	AM	AM
	18–129:E	0.58	20.53	AM	0.61	21.70	AM	17.70	22.78	11.76	M	11.28	M	AM	AM
** **	18–129:F	0.58	20.55	AM	0.61	21.72	AM	17.71	22.80	9.80	M	9.40	M	AM	AM

1. Including error range of ±2.25 years.

2. A = Adult, AA = Adolescent, CH = Child, M = Male, F = Female.

To calculate Mean Ridge Density (MRD) values, the number of ridges in 25 mm^2^ area was counted for more than one area of the print if possible, and the total mean of all the measures provided an MRD value for a print. For partial prints, a 6.25 mm^2^ area was used to measure ridge density and the count was doubled so the mean values for all prints reference the same surface area. Ridge density values *increase* due to linear shrinkage (more per surface area), so our calculations divided the density values by 2% and 6% for the archaeological sample.

Ridge breadth and ridge density data could not be taken for all prints (Table 2). Ridge breadth data could be taken for 110 prints. Five prints did not meet the standard to ensure accuracy, which requires three separate line measures across a minimum of three ridge-furrow pairs [10]. In these cases, ridge density could still be measured because the number of consecutive ridges was apparent in one or multiple 6.25 mm^2^ or 25 mm^2^ areas. As such, MRB analyses are based upon 110 prints, and the MRD analysis includes data for 115 prints.

It is worth recalling that male and female ridge breadth and ridge density values can overlap and ridge breadth measures involve a linear measure of ridges *and* furrows. Kamp et al.’s [10] Mean of Ridge-Furrow Pairs Ridge Breadth (MPRB) measures prints perpendicularly across “multiple ridge breadths simultaneously, then dividing by the number of ridge-furrow pairs” [10]. The measure assumes that ridges *and* furrows are of equal breadth. One would further assume that if ridges are “narrower” there would be more ridges in a given area. While it seems counterintuitive, there is no direct 1:1 relationship between ridge density and ridge breadth, as adolescents and adults can have ridge breadth values >0.37 mm, but the density of ridges in a given area is variable. These are related but independent measures because one is influenced by age (breadth), the other by sex (density). No one has demonstrated that ridge breadth varies predictably with sex, or that ridge density varies predictably with age. Individuals with lower ridge breadth values can have higher ridge density (a young female) or lower ridge density (a young male). Likewise, individuals with higher ridge breadth values can have higher ridge density (an adult female) or lower ridge density (an adult male).

**Fig 2:**
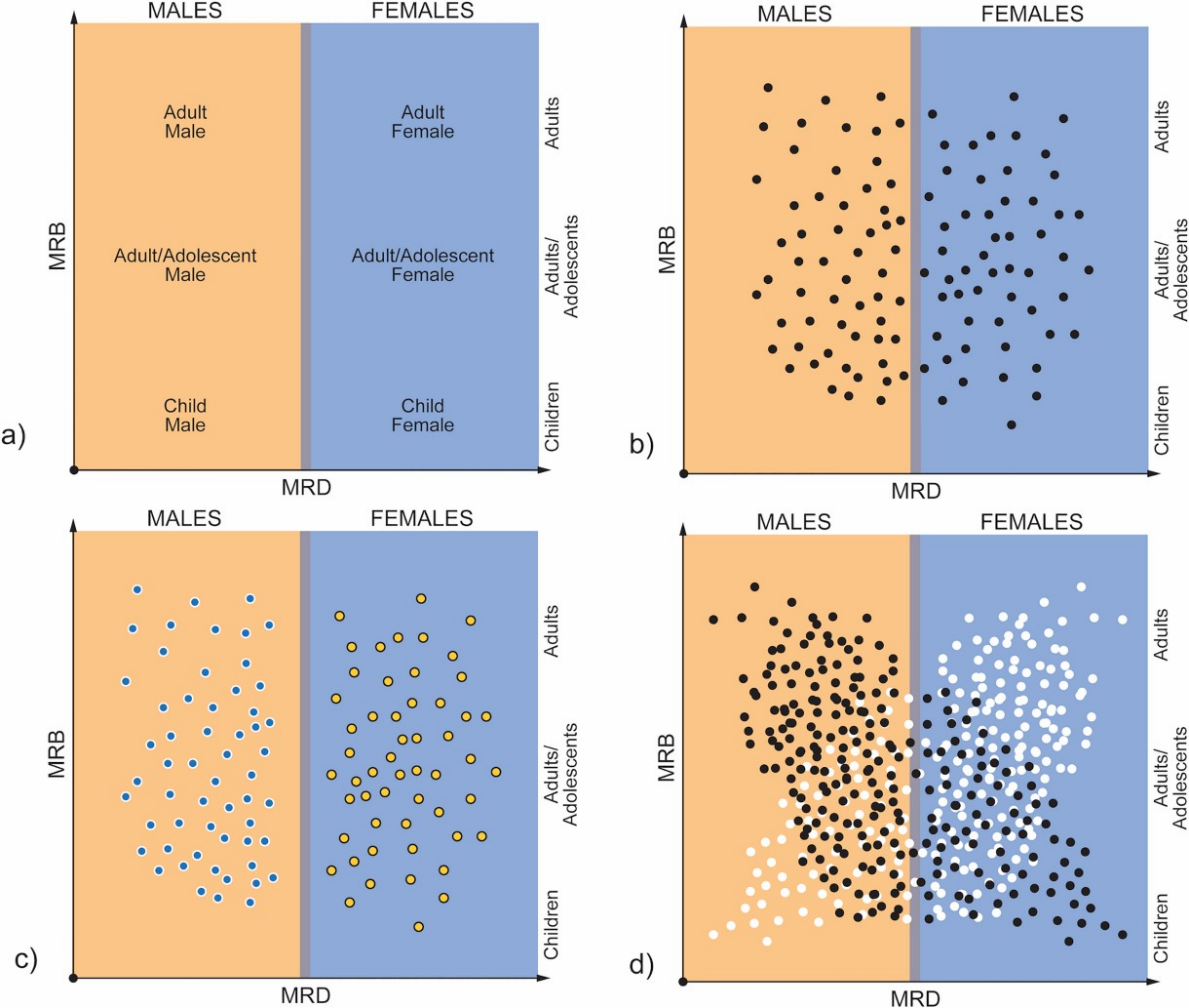
Hypothetical labour organization models based upon the distribution of Mean Ridge Bread (MRB) and Mean Ridge Density (MRD) data in the age/sex identification matrix: a) age/sex identification matrix; b) cooperative pattern; c) exclusive male (blue dots) and exclusive female (yellow dots) labour patterns; d) male dominant cooperative labour (black dots) and female dominant cooperative labour (white dots) patterns.

Our proposed *age/sex identification matrix* attempts to take this variability into account by correlating breadth and density results [3]. It further provides a means for more robust interpretation of fingerprint data because it compares both ridge breadth and ridge density data from archaeological prints against a corpus of empirical data from forensic and experimental studies. It is possible to refine interpretations by situating breadth and density data within six possible age/sex categories and to correlate the results of both methods of analysis to test whether the respective age and/or sex interpretations are supported. We suggest here that it is further possible to use the demographic profile based upon MRB and MRD distributions within the matrix to infer at least five patterns of labour organization (Fig 2): (1) if MRB and MRD data points span relatively uniformly across all six age/sex categories it is possible to infer that labour organization was *cooperative* because adults, adolescents, and possibly children are all relatively evenly represented in the demographic profile; if MRB and MRB data points fall *only* within male or female ranges across age categories it is possible to infer that labour organization was (2) *exclusively male* or (3) *exclusively female*, respectively; lastly, if more MRB and MRD data points occur within male or female ranges across age/sex categories, then it is more likely that labour organization involved (4) *male dominant cooperative labour* or (5) *female dominant cooperative labour*, respectively, as individuals of both sexes and a range of ages had some role during manufacture.

## Results

### Estimating age and sex from ridge breadth

**Fig 3:**
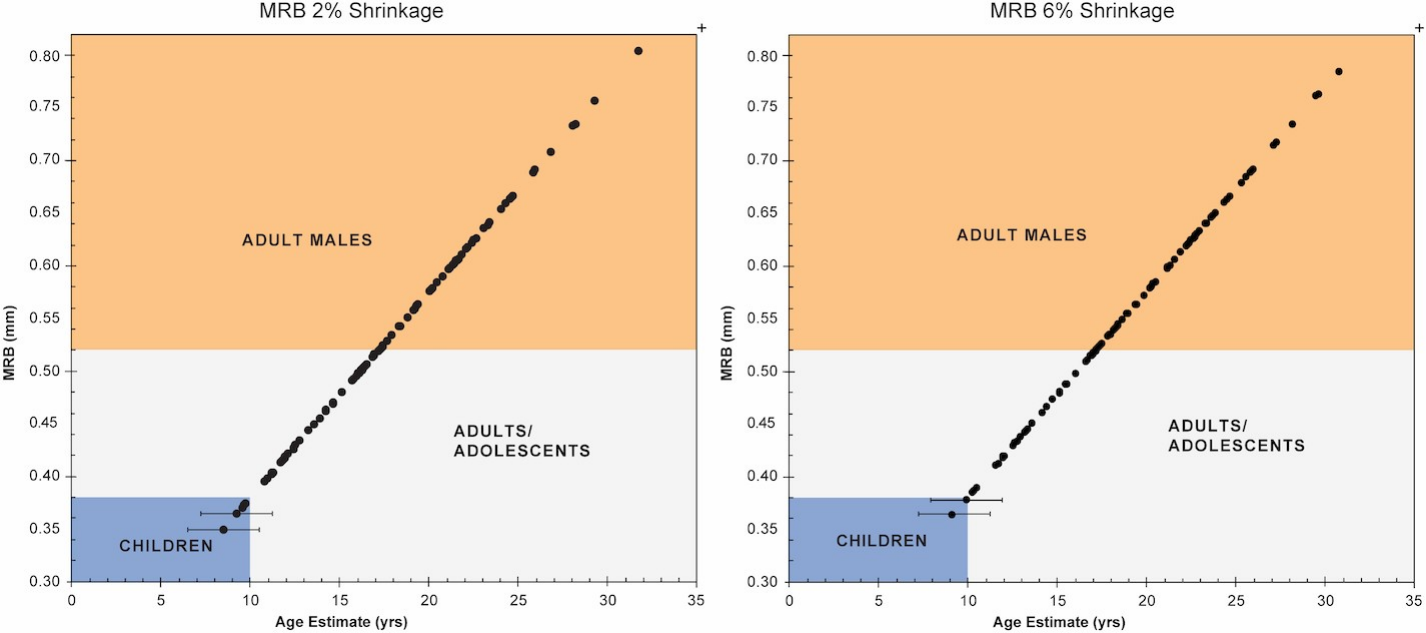
Distribution of MRB values at 2% and 6% shrinkage.

The MRB values for 110 prints at both the 2% and 6% shrinkage rates could be definitively placed in one of the three age/sex categories, including Adult Male, Adult/Adolescent, and Child (Table 3; Fig 3). The values range from 0.35 to 1.39 at 2% shrinkage and 0.36 to 1.44 at 6% shrinkage (Table 3). The 1.39 and 1.44 values represent outliers in the sample. This is a single adult male and is not shown in Fig 2 because it compresses the other results. At 2% linear shrinkage, more than half the sample are adult males (n = 60, 55%), with fewer adult/adolescents (n = 47, 43%), and three prints appear to belong to children (2.7%) although the error range extends into the Adult/Adolescent category (Fig 3). At 6% linear shrinkage, the MRB and corresponding age values increase. This results in a significant shift in the number of adult males to two-thirds of the sample (n = 72, 66%) and adult/adolescents to near a third of the sample (n = 37, 34%). The shift in the possible child prints between the low and higher shrinkage rates make it as likely that all but one of these prints were made by adolescents. In sum, the ridge breadth analysis indicates that (rounded) Adult Male:Adult/Adolescent:Child ratios of 55:43:<3 at 2% shrinkage and 66:34:<1 at 6% shrinkage characterise this set of prints. Thus, adult males and adults/adolescents dominate the age/sex categories and children are only marginally represented.

**Table 3:** Frequency of mean ridge breadth age/sex categories at 2% and 6% shrinkage.

Age/Sex	2% Shrinkage		6% Shrinkage	
	N	%	N	%
AM	60	54.5%	72	65.5%
AA	47	42.7%	37	33.6%
CH	3	2.7%	1	0.9%
Total	110	100.0%	110	100.0%

### Estimating sex from ridge density

**Fig 4:**
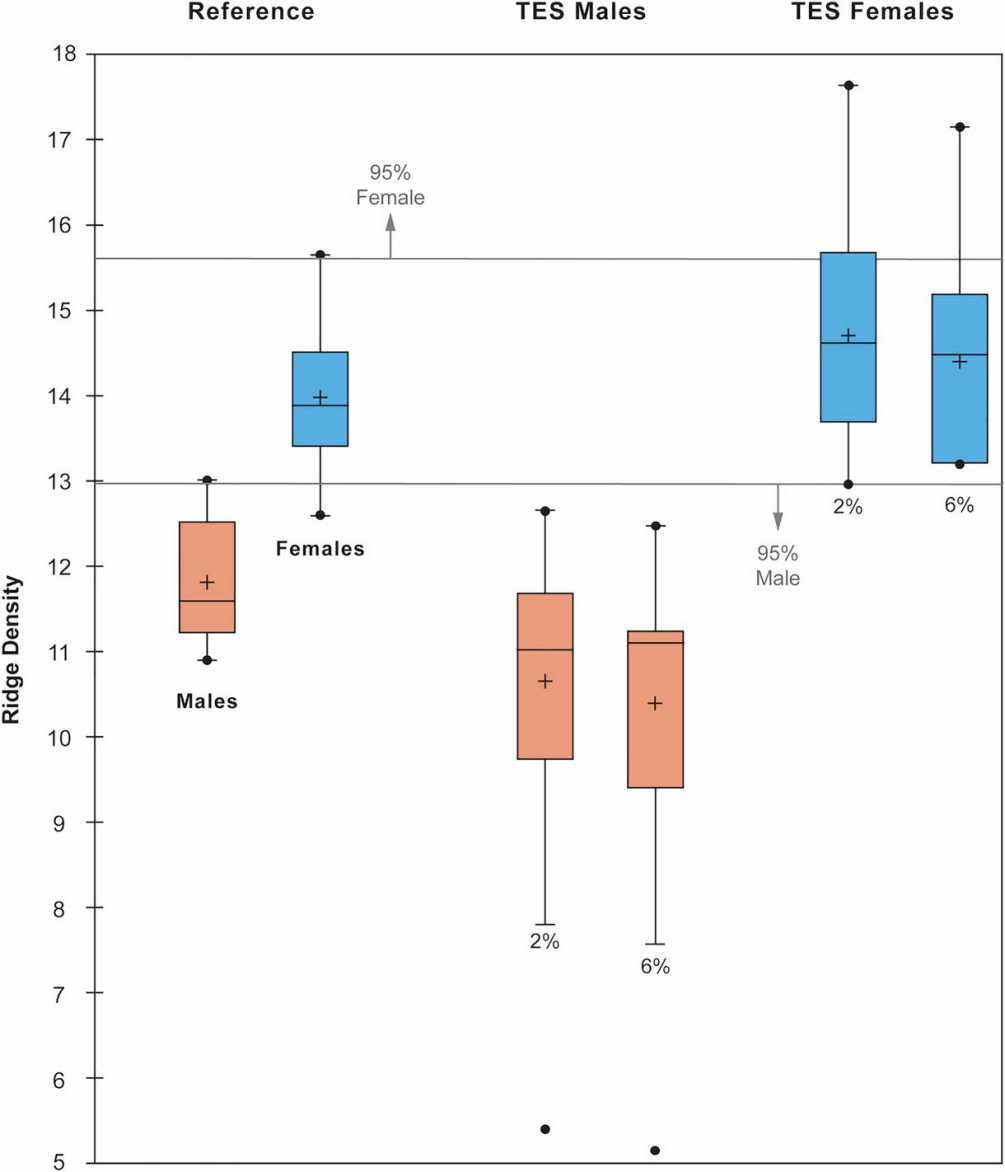
Box-plots of the male and female mean ridge density values against the reference sample.

**Fig 5:**
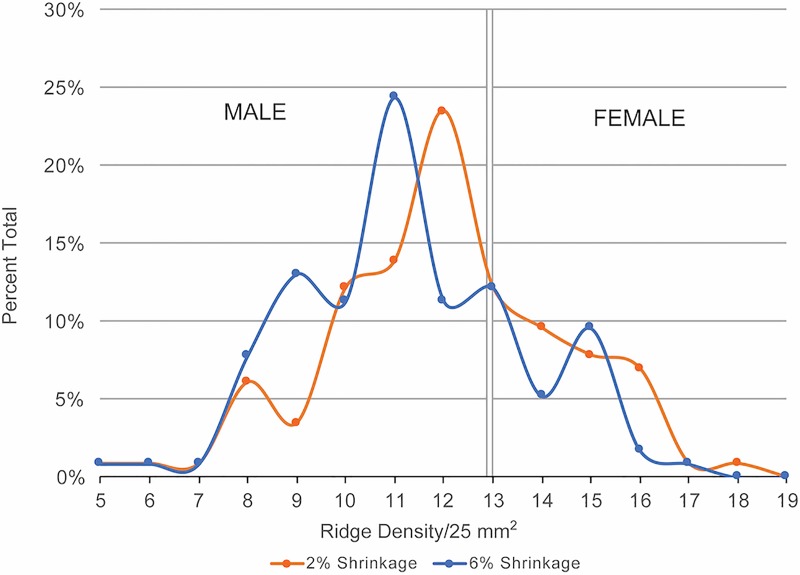
Proportion of male to female prints at the 2% and 6% shrinkage rates based on ridge density data.

The value of ridge density data is that it can help infer a print-maker’s sex from a single value when compared against a distribution of values known to be associated with either sex. In the first instance, the prints in this sample span density values from ≥5 to <19 ridges/25 mm^2^ (Table 4, Fig 4). Those values below 13/25 mm^2^ have a 95% probability of being male. One group of values from the EB III prints fall below this line, associating them with males. The higher values span ≥13–18 mm^2^, thus associating them with females. Nine prints reach the 95% confidence threshold of 15.6 ridges/25mm^2^ at 2% shrinkage and three at 6% shrinkage. When plotted, the proportions of male and female prints do not show a bimodal pattern at 2% and not 6% shrinkage because male prints dominate the sample (Fig 5). The 31 prints with values between ≥13–15 ridges/25mm^2^ after accounting for 6% shrinkage are only marginally above the high confidence threshold for identifying females. Nevertheless, because none of the 31 prints fall strictly below 13/25 mm^2^, there is a strong statistical likelihood for a respective 62:38 ratio of male to female prints at 2% shrinkage and a 70:30 ratio at 6% shrinkage. These ratios show a lower number of females than would be assumed from the adult male to adult/adolescent ratios arrived at through ridge breadth analysis (55:43 at 2%, 66:34 at 6%). While these ridge density results appear to confirm that more males made prints than females, the density values between ≥13–15.6 ridges/25mm^2^ demonstrate why it is important to consider both ridge breadth and ridge density data when interpreting prints, as there is a lower probability that these 31 prints could belong to males if they have correspondingly high ridge breadth values.

**Table 4:** Summary of sex categories from ridge density analysis of prints.

	Shrinkage				
MRD (≥mm)	2%		6%		Sex
	N	% Total	N	% Total	
5.0	1	0.9%	1	0.9%	MALES
6.0	1	0.9%	1	0.9%	
7.0	1	0.9%	1	0.9%	
8.0	7	6.1%	9	7.8%	
9.0	4	3.5%	15	13.0%	
10.0	14	12.2%	13	11.3%	
11.0	16	13.9%	28	24.3%	
12.0	27	23.5%	13	11.3%	
13.0	14	12.2%	14	12.2%	FEMALES
14.0	11	9.6%	6	5.2%	
15.0	9	7.8%	11	9.6%	
16.0	8	7.0%	2	1.7%	
17.0	1	0.9%	1	0.9%	
18.0	1	0.9%			
**Sex**					
M	71	61.7%	81	70.4%	
F	44	38.3%	34	29.6%	
Total	115	100.0%	115	100.0%	

### Identification matrix results

**Fig 6:**
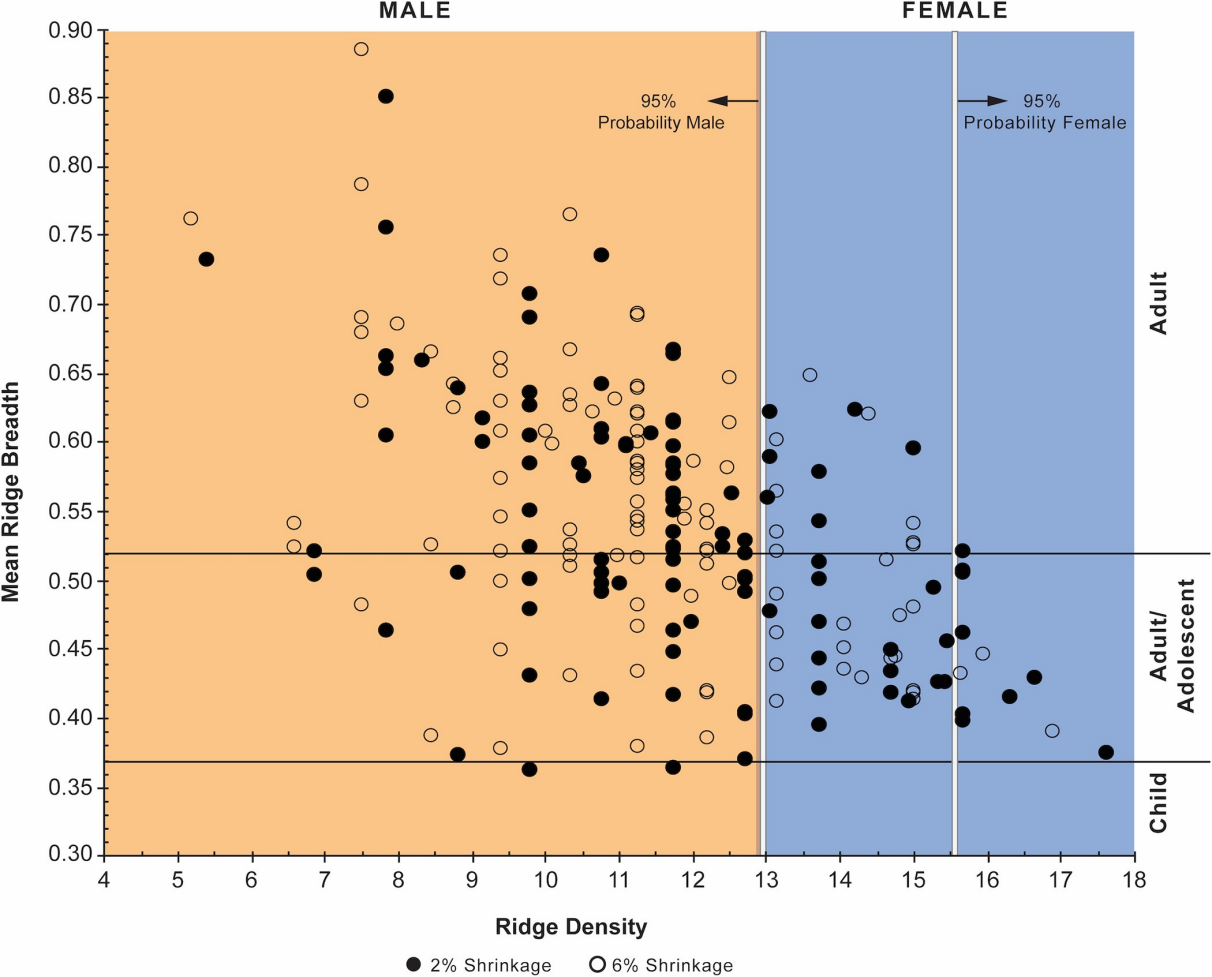
Cross-plot of mean ridge breadth and ridge density data in the identification matrix.

The total sample could be categorized into an age, sex, or age/sex combined category (Table 5). For the reasons outlined earlier, ridge breadth and ridge density data could not be taken for all prints. The combined MRB and MRD data do allow us to discuss two sets of results based on how definitive the demographic classification is: those results that fall into a definitive age, sex, or age/sex combined category, and those that fall within a non-definitive category, such as Adult/Adolescent Female, or Child. Table 6 provides the frequency of prints in each demographic category for the total sample, and Fig 6 illustrates the MRB and MRD data for the prints that have both data points at each shrinkage rate.

**Table 5:** All inferred age, sex or combined age/sex categories based upon combined MRB and MRD data for 2% and 6% shrinkage rates relative to the total analysable sample (n = 113 prints).

Categories	2% Shrinkage		6% Shrinkage	
	N	% Total	N	% Total
Adult Male	51	45.1%	61	54.0%
Adult/Adolescent Male	22	19.5%	18	15.9%
Adult Female	9	8.0%	10	8.8%
Adult/Adolescent Female	24	21.2%	19	16.8%
Child Male	2	1.8%		0.0%
Child*	1	0.9%	1	0.9%
Female	1	0.9%	1	0.9%
Male	3	2.7%	3	2.7%
Total	113	100.0%	113	100.0%

**Table 6:** Definitive and non-specific age, sex and age/sex categories based upon combined MRB and MRD data for 2% and 6% shrinkage rates.

All Age/Sex Categories	2% Shrinkage			6% Shrinkage		
	N	% Category	% Total Sample	N	% Category	**% Total Sample**
**Definitive Categories**				** **		
***Age***						
Adult	60	55.0%	53.1%	71	65.1%	62.8%
Adult/Adolescent	46	42.2%	40.7%	37	33.9%	32.7%
Child	3	2.8%	2.7%	1	0.9%	0.9%
Total	109	100.0%	96.5%	109	100.0%	96.5%
***Sex***						
Male	78	69.6%	69.0%	82	73.2%	72.6%
Female	34	30.4%	30.1%	30	26.8%	26.5%
Total	112	100.0%	99.1%	112	100.0%	99.1%
***Age and Sex***						
Adult Male	51	47.2%	45.1%	61	56.5%	54.0%
Adult/Adolescent Male	22	20.4%	19.5%	18	16.7%	15.9%
Adult Female	9	8.3%	8.0%	10	9.3%	8.8%
Adult /Adolescent Female	24	22.2%	21.2%	19	17.6%	16.8%
Child Male	2	1.9%	1.8%			
Total	108	100.0%	95.6%	108	100.0%	95.6%
**Non-specific Age/Sex Categories**						
Adult/Adolescent Female	24	51.1%	21.2%	19	40.4%	16.8%
Adult/Adolescent Male	22	46.8%	19.5%	18	38.3%	15.9%
Child	1	2.1%	0.9%	1	2.1%	0.9%
Total	47	100.0%	41.6%	38	80.9%	33.6%

Age can be inferred for 96.5% of the sample at each respective shrinkage rate (Table 6). Adults dominate the age categories at both 2% shrinkage (55%) and 6% shrinkage (65%). Adults/adolescents comprise 42% or 34% of the age categories at the respective 2% and 6% shrinkage rates. Higher shrinkage increases MRB values and decreases MRD values resulting in an overall increase in the number of adults. The child prints therefore shift from being likely at 2% shrinkage (3% of total) to two having a greater probability of being made by an adolescent at 6% shrinkage. At either rate, adults and adolescents were the primary handlers of EB III pottery, together composing 97–99% of the prints identified in this sample.

All except three prints in the sample could be identified to sex (97.4% of total). The classification is slightly altered with a change in shrinkage rate. Males (70%, 73%) outnumber females (30%, 27%) at respective 2% and 6% shrinkage rates. Male to female ratios of 70:30 and 73:24 indicate a uniformly larger number of males than were identified independently in the MRB (55:43, 66:37; Table 3) or MRD (62:38, 70:30; Table 4) analyses. This is most likely because a significant number of males are actually represented in the ridge breadth adult/adolescent category and they become more visible when MRB is considered and at higher shrinkage rates.

When age and sex are combined, a significant proportion of the sample could be definitively identified (95.6%) (Table 6). At either shrinkage rate, Adult Males (47%, 57%) and Adult/Adolescent Males (20%, 17%) compose the majority of age/sex categories relative to Adult Females (8%, 9%) and Adult/Adolescent Females (22%, 18%). These data suggest that men and teenage boys together (68%, 73%) handled pottery more regularly than women and teenage girls (29%, 26%). The prospective child prints seem entirely incidental in this light.

The non-definitive categories make up 42% and 34% of the combined data at the respective 2% and 6% shrinkage rates (Table 6). In these categories, the identification of Adult/Adolescent Females (21% to 17%) and Adult/Adolescent Males (20% to 16%) decrease in the overall sample at the higher shrinkage rate. Here again we see the effect of a higher shrinkage rate resulting in an increase in more definitively identified adults, and, in particular, adult males. There are limits to this effect, however. We have shown that shrinkage would have to be extreme (15% or more) to shift a print from the lowest values for the Adult/Adolescent category in our scheme securely into the Adult Male category [3]. Therefore, the observed increase in adults appears to be separating out late adolescents from adults that are obscured in the MRB analyses. Following from the definitive age/sex identifications above, the proportions of non-definitive age/sex categories supports the idea that a greater proportion of adolescent boys and adult men practiced the craft relative to adolescent girls and adult women.

### Application of prints during manufacturing

Prints can accrue on vessels at four stages of manufacture: during shaping, plastic decoration, post-drying decorative treatments (slips, washes, burnishing, polishing, etc.), and during post-firing treatments (glazing, plastering, resins, etc.). Since the vessels we examined are undecorated, we can be sure that prints were not set during the stage of plastic decoration when the clay was still wet or leather hard. Further, there would be little reason to touch drying vessels and it is unlikely that these prints were set during that stage. Consequently, prints were placed on this sample of vessels at two stages of manufacture: when the clay was in a plastic state during shaping and handling them afterwards, and when applying lime-plastering during the post-firing stage.

**Fig 7:**
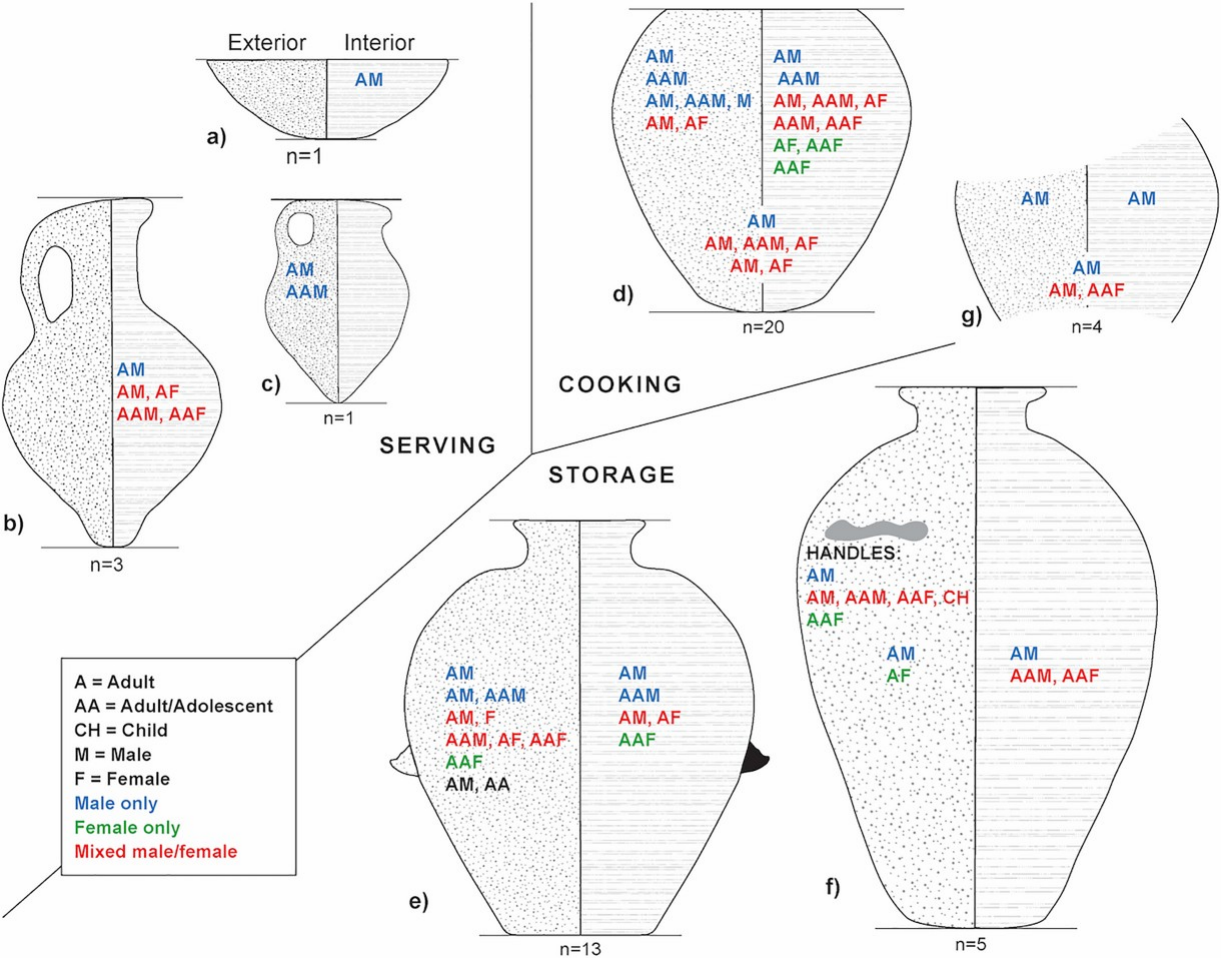
**The location and age/sex of prints represented on the vessels classes examined in the study: A) bowl, B) jug, C) juglet, D) holemouth jar, E) jar, F) wavy-handled jar, G) closed vessel.** The exterior of vessels is depicted on the left-hand side of each form and the interior on the right-hand side. Age/sex categories in the middle of the interior and interior are prints that could not be confidently assigned to either surface. The numbers below each form are the number of vessels analysed.

For prints where ridge breadth and density could be determined (n = 113), most occur on the interior of vessels (n = 50, 45%). Fewer were identified on the exterior surface (n = 32, 28%), and 11 (10%) on handles or at handle joins (interior and exterior) of wavy-handled jars. Due to the lack of curvature of some lower-body sherds (see Fig 7), the location of 20 prints (18%) on holemouth or closed vessels could not be determined with confidence. As expected, males dominate prints on each vessel type relative to females, adult/adolescents, and children (Table 7, Fig 7). Male prints are also only found on the single bowl and juglet in our sample. Otherwise, multiple prints of both sexes (but usually combinations of older and younger prints) occur on most vessel types. In instances where multiple prints occur on the interior and exterior of vessels, adults and adolescents of both sexes usually occur together. The co-occurrence of prints of different individuals on the same vessel requires explanation.

**Table 7:** Total percent of vessel types made males, females, adults/adolescents and children using identification matrix data.

	Males					Females				Children
Vessel type	AM	AAM	CHM	M	% of Type	AF	**AAF**	**F**	**% of Type**	**% of Type**
**2% Shrinkage**										
Bowl	100.0%				100.0%					
Holemouth	38.5%	23.1%	2.6%	7.7%	71.8%	12.8%	15.4%	0.0%	28.2%	
Jar	41.2%	14.7%	2.9%		58.8%	5.9%	32.4%	2.9%	41.2%	7.1%
Wavy handled jar	53.8%	15.4%			69.2%	7.7%	23.1%	0.0%	30.8%	
Jug	57.1%	14.3%			71.4%	14.3%	14.3%	0.0%	28.6%	
Juglet	60.0%	40.0%			100.0%					
Closed vessel	53.8%	23.1%			76.9%		23.1%	0.0%	23.1%	
Total	45.5%	19.6%	1.8%	2.7%	69.6%	8.0%	21.4%	0.9%	30.4%	0.9%
**6% Shrinkage**										
Bowl	100.0%				100.0%					
Holemouth	51.3%	20.5%		7.7%	79.5%	7.7%	12.8%		20.5%	
Jar	44.1%	14.7%			58.8%	14.7%	23.5%	2.9%	41.2%	
Wavy handled jar	61.5%	15.4%			76.9%	7.7%	15.4%		23.1%	7.1%
Jug	57.1%	14.3%			71.4%	14.3%	14.3%		28.6%	
Juglet	60.0%	40.0%			100.0%					
Closed vessel	76.9%				76.9%		23.1%		23.1%	
Total	54.5%	16.1%		2.7%	73.2%	8.9%	17.0%	0.9%	26.8%	0.9%

In coil building, potters apply pressure from the interior and exterior of vessels through thinning and smoothing (using their hands and various tools) to give vessels their final form. Prints on the interior surface can result from handling vessels while wiping or smoothing the exterior surface. Prints on the exterior surface could occur while supporting the vessel to thin and smooth the interior surface or when handling them after preforming was complete and they were moved for drying. Thus, all the prints in our sample were set during the preforming operations or after those were completed. However, wavy-handled jars provide a different sequence. The prints on three wavy-handled jars occur in the curved areas of handles and on the interior body where handles were applied after the interior and exterior had been wiped. This means that handles were applied after the preform had reached its final shape. Either men or adult/adolescent females alone seem to have usually placed the handles on the jars (Table 8). However, for one vessel (18–117), the handle and the interior surface has prints of an adult man, an adult/adolescent male and female, and a child. It is very tempting to envision a young man or woman shaping the handle, a younger sibling helping secure it to the vessel, and the more experienced adult potters checking if it is well attached or repairing the join. All four must have been present when the handle was attached.

**Table 8:** Age/sex of prints on the handles and interior surface of handle joins on wavy-handled jars.

Age/Sex	Vessel			Total	% Type
	18–117	18–122	18–123		
Adult male	2		4	6	55%
Adult female				0	0%
Adult/adolescent male	1			1	9%
Adult/adolescent female	2	1		3	27%
Child	1			1	9%
Total	6	1	4	11	100%

The occurrence of multiple interior prints indicate that the hands of men, women and adolescents were inside the vessels during preforming. Fig 7 only shows the age and sex represented by prints on a vessel, not the number of each category because, as mentioned earlier, we could be identifying prints of the same individual in instances where the age and sex estimations for different prints overlap, but in other cases there are co-occurring prints of different age and sex. It is possible that the prints of different individuals were set at the same time on the interior of pots if one hand was guiding the work of another. Alternatively, different hands could have been placed on the inside of pots at different times. This is most likely in the case of holemouth jars, as they have the greatest variation in print combinations on the interior surface. The opposite pattern occurs with jars, which have the most print combinations on the exterior surface.

There are two most likely scenarios to explain the multiple prints. First, certain prints were placed when shaping vessels and others accrued during handling them after shaping was complete, either to inspect the vessels or to move them to a drying location. Second, it is reasonable to suggest that prints we observed on the interior surface resulted from gestures used to stabilize vessels during shaping or while performing surface treatments and those that occur on the exterior resulted from stabilizing or moving vessels. Prints of different individuals could occur on the interior surface when one individual took over the task of exterior finishing from another. Such a transfer would add flexibility to the work schedule and could also reflect the training of novice potters. It is again most tempting to infer the training of younger potters in the cases where there are younger and older prints on the interior of jars (e.g., 17–101, 124, 148). Further, the tendency of older prints on exteriors and younger prints on the interiors of holemouth and storage jars points to possibilities for investigating the training of potters, particularly if vessels can be found that have prints on both exterior and interior surfaces. In either scenario, it is quite difficult to distinguish whether the handling of vessels was done by males or females of different ages during the preforming stage and after it was completed. What we can conclude with some certainty is that all of the vessels with multiple prints show that older adolescents, adult females or males worked alongside younger members of the same or different sex during the shaping stage of manufacture.

## Discussion and conclusions

The forgoing analyses provides a larger, more robust sample to examine hypotheses stemming from our earlier effort to establish an interpretive method and framework for the age and sex of fingerprints on EB III pottery in the Levant. Here, we reconsider the hypotheses against this new evidence.

### Hypothesis 1. Men and women made different kinds of pottery

We contend that an absence of prints for one sex on a type of pottery indicates a division of labour by vessel function. With our smaller sample, we noted a discrepancy in prints occurring on different type of pottery: males exclusively made bowls and cooking ware and males and females made storage jars. Contrary to our previous findings, in this larger sample male and female prints are found on all but two vessels types. Only the single bowl and juglet in our sample have prints of adult and adult/adolescent males. In contrast, adults and adolescents of either sex left prints on holemouth cooking jars, jugs, and both kinds of storage jars, although adult males dominate prints on each vessel type. A larger sample of bowls and juglets may alter this result in the future. Presently, however, we can propose that the manufacture of some vessel types was restricted by age and sex.

### Hypothesis 2. The labour force used to make EB III pottery involved men and women and teenagers of either sex

As noted in the introduction, it is extremely difficult to parse out and identify the prints of biological adolescents due to rapid growth after the onset of puberty and because hands stop growing in length (but not thickness) around the age of 15. Yet, combining age and age/sex estimates provides a means to separate out adults from the adult/adolescent range, leaving it more likely that adolescents are represented in the category. Our results indicate that adolescents of either sex are implicated in the manufacture of all vessel types except bowls. However, because the ambiguity of the adolescent range, we can only say that 33–41% of the fingerprints *could* represent adolescents (Table 6). Age ranges help clarify the category. Based on matrix data, the *average* ages of individuals in the adult/adolescent category span 7–14 years, including the margin of error (see Table 2). This is precisely the age range for the onset of pubescence predicted by Gluckman and Hanson [19] for prehistoric populations with good health and food security. Men, women and teenagers of either sex did indeed comprise the labour force involved in making EB III pottery at Tell eṣ-Ṣâfi/Gath from c. 2700–2600 BCE. Further, we can propose that pottery-making was a male dominated craft that relied on the cooperative effort of women and adolescents.

### Hypothesis 3. Multiple hands were normally involved in vessel shaping and adults and teenagers had different roles in manufacture

Two-thirds of vessels in our sample (n = 31/47, 66%) have two or more prints classified in different age/sex categories. The co-occurrence of adult and possible adolescent prints on the same sherd and on the same surface strongly indicate that the hands of these different individuals touched the wet or leather-hard vessels between the time the surface was finished and before the vessels had dried to the extent that prints would not be retained.

Using coiling methods, multiple hands cannot simultaneously be on the inside of a vessel while making the preform (rough cylindrical shape). However, multiple prints could accrue on the interior surface if more than one person was involved in making the preform and the operations involved in creating the roughout (the final form), such as placing a hand on the interior and wiping or scraping the exterior surface [3]. As the prints we observed on the exterior surface were placed *after* the surface was finished and not before, the exterior prints in our sample must have been set when handling the finished roughout. Thus, we have entertained two possibilities for multiple prints on the interior and exterior of vessels: different sequential steps involved in shaping vessels were done by more than one individual, or multiple hands were involved during each sequential step in the process of training novice potters. The main problem is that, as yet, it is not possible to distinguish between the two scenarios because this requires vessels that have prints on both the interior and exterior surface. Nevertheless, we can expand on the possibility that training novices was responsible for multiple prints on the interior of vessels.

Judging from the RB age range of 10–19 years, the youngest individuals would all be pubescent and the oldest would not yet be considered biological adults. Given the broad range of cognitive abilities, dexterity and strength that span across this age range, it is more likely that those of more advanced age, experience, strength, and skill would take on a role of teaching and fostering the development of younger print-makers by involving them in certain steps of manufacture, particularly by having young adolescents assist with materials and tools, smoothing operations, and moving vessels when they were finished to be set for drying. With clear evidence that that older and younger potters of the same sex were involved in the manufacture of wares during the same manufacturing episodes, it is reasonable to infer that that older potters would be instructing younger ones in the craft.

### Hypothesis 4. A greater proportion of teenage boys learned to be potters and practiced the craft while adults, while fewer teenage girls continued to make pottery into adulthood

This hypothesis is based upon the assumption that learning normally occurred during adolescence and not adulthood. This is a rather normative assumption [51]. However, it is not universal. Many Zulu women, for instance, tend to engage in learning most aspects of potting, passively then actively, while young, but shaping is not learned or practiced until women are married and/or have had their first child [52–54]. At Tell eṣ-Ṣâfi/Gath, adolescent learning may not have been followed by every individual, but it is more likely that this data set will show a tendency towards a typical learning pattern while including some exceptions. It is possible to test the hypothesis by examining ratios of adult male:female prints to adolescent male:female prints.

The matrix data shows adult male:female ratios of 47:8 and 57:9 ratios at the 2% and 6% shrinkage rates (Table 6). By contrast, the adult/adolescent male:female ratios are 47:51 and 40:38 at the respective 2% and 6% shrinkage rates (Table 6). It would appear that young men and women were both provided the opportunity and engaging in the manufacture of pottery, but far fewer young women continued to either make pottery or be engaged in the craft in such a way that they would leave prints on vessels. More specifically, far fewer adult females were directly involved in the shaping stage of manufacture. We cannot conclude that adult and young women were less involved in all other stages of manufacture, such as acquiring and preparing clay, or firing vessels. From our singular example of plaster on a storage jar, it does not seem that adult and young women were involved in at least this particular post-firing treatment. Additionally, we have no means to examine whether women were responsible for painting vessels, such as platters, as we have found no fingerprints as yet on the vessel type. At present, however, it is clear that more adult and young men left prints on pottery and were principally engaged in the crucial stage of shaping vessels.

### Hypothesis 5. A larger sample size would demonstrate more males than females were involved in potting, but a larger proportion of prints would be identified to adult females and individuals of adolescent age

This hypothesis speaks to the effects of sample size. The potential influence of sample size can be examined by comparing the results of our original sample (n = 38 prints) with the current sample (n = 112) for which both age and sex data is available. It is only necessary to compare two sets of results, the relative proportion of males to females in each sample, and the relative proportion of adult females and adolescents. Table 9 summarizes this comparison by showing the proportion of males to females for each sample size and the relative proportion of adult females and the combined adult/adolescent categories (adult/adolescent male, female, and adult/adolescent) at each shrinkage rate.

**Table 9:** A comparison of sex and select age/sex determinations made on the pilot (n = 38) and full (n = 115) sample size of prints.

	2% Shrinkage		6% Shrinkage	
Categories	N	% Total Sample	N	% Total Sample
Sex (38 print sample)				
Male	24	63.2%	26	68.4%
Female	10	26.3%	10	26.3%
Total	34	89.5%	36	94.7%
**Sex (115 print sample)**				
Male	78	69.0%	82	72.6%
Female	34	30.1%	30	26.5%
Total	112	99.1%	112	99.1%
**Adult Female/Adolescents (38 prints)**				
Adult Female	3	7.9%	5	13.2%
Adult/Adolescent	6	15.8%	4	10.5%
Combined	9	23.7%	9	23.7%
**Adult Female/Adolescents (115 prints)**				
Adult Female	9	8.0%	10	8.8%
Adult/Adolescent	24	22.2%	19	16.8%
Combined	33	29.2%	29	25.7%

The male:female ratios show that the proportion of male prints increased from the smaller to the larger sample size (63:68% to 69:73% at 2% and 6% shrinkage) and the proportion of female prints slightly increased at 2% shrinkage in the larger sample but slightly decreased at 6% shrinkage (26:26% to 30:27% at 2% and 6% shrinkage). Thus, a larger proportion of female prints were only identified at the smaller shrinkage rate. By increasing shrinkage, a small number of female prints (n = 4) were consequently identified as male. As expected, overall, there were a larger proportion of either sex identified. However, when considering the proportion of adult females and adult/adolescents, there is a greater decrease in the number of adult/adolescent females identified in the larger sample at the 6% shrinkage rate (16% and 11% in 38 print sample, 22% and 16% in 115 print sample, at respective 2% and 6% shrinkage). The proportion of adult females increases slightly with shrinkage in the 38 print sample (8% and 13% at respective 2% and 6% shrinkage) and less so in the 115 print sample (8% and 9% at respective 2% and 6% shrinkage) (Table 6). From this comparison, we take away that, proportionally, slightly more adult/adolescents were identified in the larger sample, but the relative proportion of females and adult females does not vary significantly. This comparison is promising for refining the age and sex determination of prints. By combining the MRB and MRD results, we are able to distinguish some of the males and females in the 5% overlap in MRB and MRD values for each sex to provide a more realistic proportion of females in this population. We can be more confident that the skew towards more males in both samples reflects male dominant production arrangements that regularly included adult women and adolescents, and, for the attachment of some handles, children.

Our testing of these hypotheses provides new insights into the manufacture of wares, learning strategies, and the division of labour in an EB III community of potters that spanned several generations. The reference models, methods and hypotheses have utility for research on different periods within and beyond the study area. However, unlike areas where fingerprint research has not been conducted, we do have the advantage of examining potential patterns of labour divisions regionally.

Our characterisation of male dominant production arrangements during the EB III at Tell eṣ-Ṣâfi/Gath presents a very different situation than has been inferred for northern Mesopotamia, where Sanders [16] argued that men exclusively made pottery after the emergence of urbanism and the state in northern Mesopotamia. The results of Sanders’ study and our study are incommensurable. Several significant issues present obstacles to comparison. Sanders only analysed ridge density in the Tell Leilan and other collections he examined. Thus, it is only possible for us to compare sex. He also did not propose a confidence level for the results, so there is no way of knowing the *likelihood* that Sanders’ values represent males or females. This is important because ridge density values for males and females do overlap, and values in his sample fall within the overlapping range of male and female prints in the reference sample he used [from 15]. In examining Sanders’ [16] provided data (Table 4) more closely, and using our 95% probability cut-off ranges for males (<12.99 ridges/25mm^2^) and females (>15.6 ridges/25mm^2^), it is more likely that one print is male, seven are female, and three are definitely female. Thus, almost all (n = 10, 91%) of the “state-era” set of prints reported by Sanders were probably made by female potters in rural areas. While problematic, the “state-era” analysis by Sanders’ raises the intriguing possibility that rural pottery production many not be organised in the same fashion as urban production, and future analyses must also pay attention to prints found on pottery from towns, villages, camps, and caves.

Given that our analysis of two sample sets from Tell eṣ-Ṣâfi/Gath present consistent demographic evidence for male dominant cooperative production, we now have a basis to investigate whether prints from other EB III urban centres in the Levant show the same pattern and whether the organisation of pottery production across the region could be differently organized than in northern Mesopotamia. The latter hypothesis would align with the general observation that city-states in Mesopotamia and the Levant took different forms. We should not, then, expect crafts production to be organised in precisely the same ways. We may well be observing considerable variation in the organisation in crafts production arrangements between primary (Mesopotamia) and likely secondary (Levant) states that cannot easily be cast under the monolith of “craft specialisation” despite the overwhelming evidence that this was a period of profound social change in the Levant.
